# Different Forms of Regulated Cell Death in Type-2-Diabetes-Mellitus-Related Osteoporosis: A Focus on Mechanisms and Therapeutic Strategies

**DOI:** 10.3390/ijms26094417

**Published:** 2025-05-06

**Authors:** Chenchen Li, He Gong, Peipei Shi, Shuyu Liu, Qi Zhang

**Affiliations:** 1 Key Laboratory of Biomechanics and Mechanobiology, Ministry of Education, National Medical Innovation Platform for Industry-Education Integration in Advanced Medical Devices, School of Biological Science and Medical Engineering, Beihang University, Beijing 100191, China; lichenchen0020@buaa.edu.cn (C.L.); bme-peipeishi@buaa.edu.cn (P.S.); liushuyu@buaa.edu.cn (S.L.); zhangqii@buaa.edu.cn (Q.Z.); 2 Medical Engineering & Engineering Medicine Innovation Center, Hangzhou International Innovation Institute, Beihang University, Hangzhou 311115, China; 3 Key Laboratory of Innovation and Transformation of Advanced Medical Devices, Ministry of Industry and Information Technology, Beijing 100191, China

**Keywords:** type 2 diabetes mellitus, diabetic osteoporosis, cell death, cell signaling

## Abstract

Type 2 diabetes mellitus (T2DM) is a chronic metabolic disorder with a high prevalence and challenging treatment options. It significantly affects the function of various organs, including bones, and imposes substantial social and economic costs. Chronic hyperglycemia, insulin resistance, and abnormalities in glucolipid metabolism can lead to cellular damage within the body. Bone dysfunction represents a significant characteristic of diabetic osteoporosis (DOP). Recent studies confirm that cell death is a critical factor contributing to bone damage. Regulated cell death (RCD) is a highly controlled process that involves numerous proteins and specific signaling cascades. RCD processes, including apoptosis, autophagy, necroptosis, pyroptosis, ferroptosis, and cuproptosis, may be linked to the dysfunction of bone cells in T2DM. In this review, the cell death types of bone cell populations during the pathogenic process of DOP were explored, and the link between cellular RCD processes and the pathogenesis of DOP was further explored. In addition, the research progress on targeting RCD for DOP was summarized in this paper. This may provide a foundation for additional explorations and drug development, as well as new therapeutic concepts for the clinical management of DOP.

## 1. Introduction

Diabetes mellitus (DM) is a chronic metabolic disease characterized by hyperglycemia, causing serious threats to human health and imposing a substantial societal burden [1]. The classification of DM includes four main categories: type 1 diabetes mellitus (T1DM), which is characterized by autoimmune-induced destruction of pancreatic β-cells and an absolute lack of insulin; type 2 diabetes mellitus (T2DM), marked by insulin resistance and a decline in pancreatic β-cell function; gestational diabetes mellitus (GDM), which occurs during pregnancy and typically resolves after childbirth; and specific types of DM, which encompass other less common forms of the disease [2]. The International Diabetes Federation (IDF) Diabetes Atlas has reported that the global prevalence of diabetes among people aged 20–79 years was approximately 10.5% (540 million) in mid-2021, projected to rise to 12.2% (780 million) by 2045, with T2DM constituting 90% of the overall prevalence [3]. Although both T1DM and T2DM patients face an increased risk of fractures, the pathophysiologic mechanisms underlying skeletal effects in T2DM are more complex [4]. Unlike T1DM patients, who typically experience a significant decrease in bone mineral density (BMD), BMD changes in T2DM patients are not uniform and may even be elevated to varying degrees. This can lead to an underestimation of fracture risk in T2DM patients in clinical practice [5]. Therefore, understanding the mechanisms by which T2DM causes bone damage is particularly crucial. In recent years, T2DM is regarded as a primary contributor to impaired bone remodeling and hence osteoporosis [6]. Diabetic osteoporosis (DOP) is a complication of DM that is influenced by the microenvironment of T2DM and is characterized by structural abnormalities of bone tissue and compromised bone quality [7]. The significant rise in the global diabetic population indicates that DOP will pose a serious threat to the quality of life for such people [8]. Bone remodeling is a dynamic process that involves bone resorption and formation, significantly contributing to bone strength and integrity. The processes require the coordination of multiple cell types, including osteoblasts, osteoclasts, osteocytes, and bone marrow mesenchymal stem cells (BMSCs). They are influenced by various physiological and pathological factors [9]. Normal treatments for DOP primarily include pharmacological agents that enhance bone formation or suppress bone resorption. A few drugs utilized for the treatment of DM exhibit a protective effect on bone health. Nonetheless, effective clinical therapies for DOP remain insufficient, largely due to the intricate pathogenesis of the condition, which includes glycolipotoxicity, metabolic reprogramming, insulin resistance, oxidative stress, and pro-inflammatory cytokines [10]. These factors may contribute to cell death in bone tissue, thereby inducing bone damage. A thorough comprehension of the various modes of cell death in DOP will facilitate the identification of new targets to improve DOP treatment.

**Figure 1:**
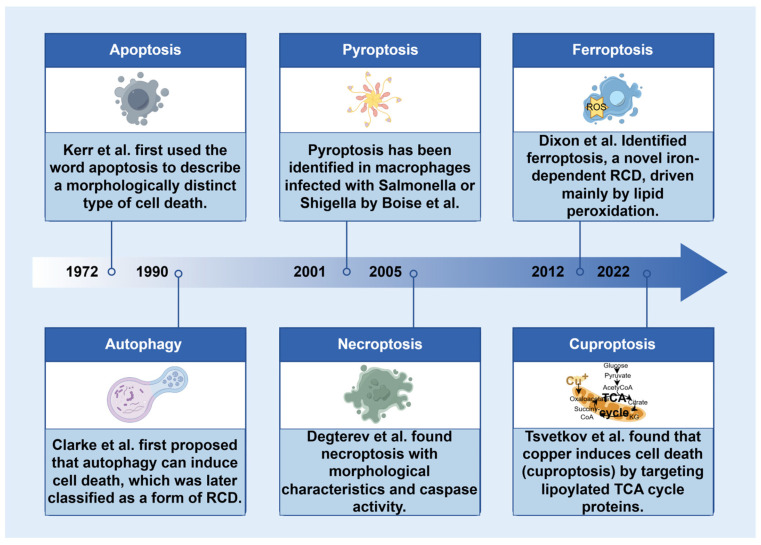
Timeline of the terms used in cell death research. RCD, regulated cell death; TCA, tricarboxylic acid.

Cell death is an irreversible process induced by exogenous or endogenous substances [11]. Cell death can be divided into two categories based on the 2018 classification provided by the Nomenclature Committee on Cell Death (NCCD), i.e., regulated cell death (RCD) and accidental cell death (ACD) (Figure 1) [12]. ACD is a biologically uncontrolled cell death induced by severe physical, chemical, or mechanical injury processes. RCD, on the other hand, is a tightly regulated process that occurs in a physiological state and involves a cascade of signals [13]. RCD can be categorized into two groups based on morphological changes, i.e., apoptosis and autophagy, neither of which result in cell rupture or the release of cellular contents [14]. Apoptotic cells can generate apoptotic bodies, and dead cells generated by apoptosis are quickly engulfed by macrophages for degradation [15]. Autophagy is a lysosome-mediated process that eliminates molecules and subcellular elements, including nucleic acids, proteins, lipids, and organelles [16]. Mild autophagy serves as a protective mechanism for cells, whereas excessive autophagy can result in cell death [17]. Neither type of RCD induces a pericellular inflammatory response. The second type of RCD includes pyroptosis and necroptosis, characterized by cell rupture and subsequent release of significant quantities of proinflammatory factors. Pyroptosis is a type of regulated cell death initiated by the activation of inflammasomes, such as the NLRP3 inflammasome. Necroptosis typically occurs as a consequence of the inhibition of the apoptotic pathway [18]. Ferroptosis and cuproptosis have recently emerged as significant forms of RCD. Ferroptosis is an iron-dependent RCD caused by the accumulation of lipid peroxides and the reduced expression of antioxidant factors such as glutathione (GSH) and glutathione peroxidase 4 (GPX4) [19]. The main mechanism of cuproptosis requires the direct interaction of copper with the fatty acylation-related components of the tricarboxylic acid (TCA) cycle, resulting in the disruption of the TCA cycle. This disruption subsequently triggers proteotoxic stress and induces cell death [20].

**Figure 2:**
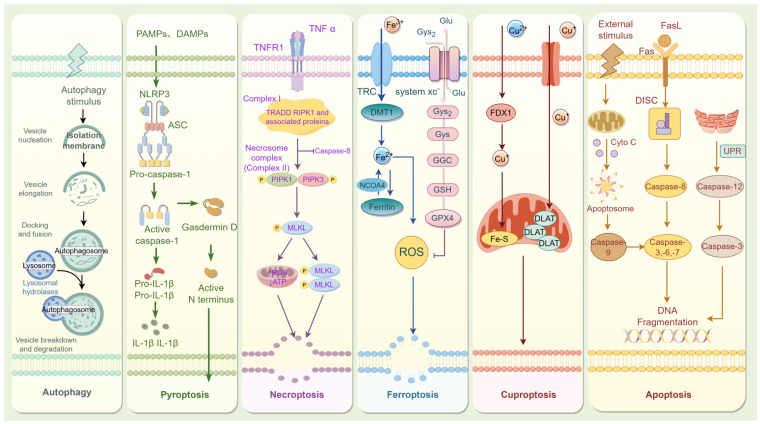
Schematic illustration of regulated cell death. Abbreviations: ASC (apoptosis-associated speck-like protein containing a CARD); ATP (adenosine triphosphate); Cyto C (cytochrome C); DAMPs (damage-associated molecular patterns); DMT1 (divalent metal transporter 1); DLAT (dihydrolipoamide S-acetyltransferase); DISC (death-induced signaling complex); FDX1 (Ferredoxin1); Fas (tumor necrosis factor receptor superfamily, member 6); FasL (Fas Ligand); Glu (glutamine); Gys2 (glycogen Synthase 2); Gys (cysteine); GGC (γ-Glutamylcysteine); GSH (glutathione); GPX4 (glutathione peroxidase 4); IL-1β (interleukin-1beta); IL-18 (interleukin-18); MLKL (mixed lineage kinase domain-like protein); NLRP3 (NOD-like receptor protein 3); NCOA4 (nuclear receptor coactivator 4); PAMPs (pathogen-associated molecular patterns); PIPK1 (receptor-interacting protein kinase 1); PIPK3 (receptor-interacting protein kinase 3); ROS (reactive oxygen species); TNF-α (tumor necrosis factor-alpha); TNFR (tumor necrosis factor receptor); TRC (transferrin receptor protein 1); UPR (unfolded protein response).

RCD is a critical biological process that is involved in a diverse array of physiopathologic conditions. Under physiological conditions, RCD eliminates unwanted or abnormal cells to preserve homeostasis in the body. In contrast, in pathological environments, dysregulated RCD may result in excessive cell death, disrupting intercellular coordination and homeostasis, which can accelerate the disease process [21]. The elevated glucose levels in T2DM patients and animal models represent a significant pathological characteristic [5,22]. Prolonged exposure of bone tissues to a high-glucose environment impacts cellular activity, resulting in metabolic disorders and abnormal cellular function, ultimately leading to cell death [4]. To investigate the specific mechanisms by which T2DM induces cellular dysfunction and RCD abnormalities in bone tissue, the researchers simulated the T2DM microenvironment using high glucose and established in vitro cellular models [23]. With the advancement of disease pathophysiology, an increasing number of T2DM causative factors have been incorporated into in vitro models, including advanced glycosylation end products (AGEs), hyperlipidemia, and inflammatory factors [24,25,26]. Recent studies have increasingly focused on the effects of the T2DM microenvironment on the RCD process in bone tissue cells. These studies aim to elucidate the roles of various RCD mechanisms in DOP and to identify potential therapeutic strategies for alleviating DOP by targeting RCD-related pathways (Figure 2). The impact of RCD on the pathogenesis of DOP may offer a novel strategy for therapeutic intervention. This review focuses on the mechanisms of cell death in DOP and the pharmacological and natural compounds used to treat osteoporosis by targeting these pathways, aiming to generate novel insights for therapeutic strategy development in DOP.

## 2. Apoptosis

### 2.1. Overview of Apoptosis

Apoptosis is a homeostatic and non-inflammatory mode of RCD, induced by apoptotic caspases and modulated by the expression levels of pro- and anti-apoptotic Bcl-2 family members [27]. Apoptotic cells exhibit morphological features including cell shrinkage, fragmentation into membrane-bound apoptotic bodies, and swift phagocytosis by adjacent cells (Table 1) [28]. Apoptosis is regulated by caspases. Activation of caspases by apoptotic signals triggers a cascade of reactions that culminate in irreversible apoptosis [29]. The primary regulatory pathways of apoptosis are categorized into three types, i.e., the exogenous pathway, which is regulated by caspase-8 (death receptor pathway); the endogenous pathway, regulated by caspase-9 (mitochondrial pathway); and the endoplasmic reticulum (ER) stress pathway (Figure 2) [30].

### 2.2. Apoptosis in DOP

Apoptosis, the first recognized type of regulated cell death, has been thoroughly investigated in relation to diabetic complications. The etiology of DOP is multifactorial, including hyperglycemia, inflammation, oxidative stress, mitochondrial dysfunction, endoplasmic reticulum stress, and cell death [31]. In DOP, hyperglycemia and metabolic disturbances result in the early accumulation of AGEs, which promote the generation of reactive oxygen species (ROS), disrupt mitochondrial function, and ultimately activate both exogenous and endogenous apoptotic pathways [32]. In diabetic animal models, increased apoptosis rates have been observed in both bone tissue and isolated cultured cells [33]. Elevated glucose levels stimulated the secretion of matrix metalloproteinases from osteoblasts through the regulation of cell-cycle- and apoptosis-related factors. This modulation impacted cell proliferation and migration, enhanced apoptosis, and ultimately resulted in impaired osteogenic function [34]. Animal experiments have similarly demonstrated the role of apoptosis in DOP. A notable increase in TdT-mediated dUTP nick-end labeling, (TUNEL)-positive cells was observed in the bone sections of diabetic mice, suggesting that diabetes-induced apoptosis in bone contributes to bone loss, ultimately resulting in lowered mechanical properties of the bone [35].

In T2DM rats induced by high-fat diet (HFD) and streptozotocin (STZ), there was a significant reduction in the expression levels of the anti-apoptotic B-cell lymphoma 2 (Bcl-2) and B-cell lymphoma-extra large (Bcl-xL) mRNA in the tibiae, accompanied by a significant increase in the expression levels of the pro-apoptotic Bcl-2-associated X (Bax) and Bcl-2 homologous antagonist/killer (Bak). It was observed that activities of caspase-8, caspase-9, and caspase-3 were significantly elevated, while bone formation indices such as alkaline phosphatase (ALP), osteoprotegerin (OPG), and receptor activator of nuclear factor-kappa B ligand (RANKL) were significantly reduced in the tibiae of T2DM rats. These results show an activation of the apoptotic pathway and a reduction in osteogenesis in bone tissues under the pathological condition of T2DM. Further in vitro mechanistic study using the SaoS-2 cell line demonstrated that high glucose activated pro-apoptotic factors while simultaneously inhibiting the expression of anti-apoptotic factors. This dual effect led to decreased osteogenic activity and induced apoptosis. Consequently, the activity of ALP and the expression level of runt-related transcription factor 2 (Runx2) were reduced, whereas the expression levels of tumor necrosis factor-alpha (TNF-α), interleukin-1beta (IL-1β), cyclooxygenase 2 (COX2), and matrix metalloproteinases-14 (MMP-14) in osteoblasts were increased [36]. In addition to influencing the expression of apoptotic pathways represented by the caspases, high glucose can also induce apoptosis by regulating endoplasmic reticulum stress. High glucose has been demonstrated to induce post-translational activation of eukaryotic translation initiation factor 2α (eIF2α) via increased calcium flux and upregulation of binding immunoglobulin protein (BiP, a major ER chaperone). This results in the post-translational activation of activating transcription factor 4 (ATF4) and the upregulation of C/EBP homologous protein (CHOP), ultimately causing osteoblast apoptosis due to endoplasmic reticulum stress. The use of melatonin reduced endoplasmic-reticulum-stress-induced apoptosis by affecting the PERK-eIF2α-ATF4-CHOP axis, thereby securing osteoblasts from damage caused by high glucose [37]. In T2DM, the formation and accumulation of AGEs are accelerated, and the aberrant elevation of AGEs and their receptor RAGE in bone tissue may be associated with the development of DOP [38]. When the human osteoblast cell line hFOB1.19 was cultured in the presence of different concentrations of AGEs, apoptosis was induced in a caspase-dependent manner. High concentrations of AGEs significantly increased apoptosis in osteoblasts and upregulated the mRNA levels of RANKL via the RAGE/Raf/MEK/ERK signaling pathway. Conversely, the mRNA levels of ALP, osteocalcin (OCN), and OPG were downregulated. These alterations collectively impaired the bone remodeling functions of hFOB1.19 cells [39]. Additionally, clinical evidence revealed a significant positive correlation between AGEs levels and the proportion of TUNEL-positive osteoblasts in human vertebral bone tissue (r = 0.72). Further in vitro studies demonstrated that AGEs could induce apoptosis by activating ER-stress-related proteins, such as GRP78, IRE1α, and c-Jun, in mouse osteoblasts (MC3T3-E1). The activity and function of osteoblasts were improved with the application of ER stress inhibitors [40]. The aforementioned findings indicate that the T2DM microenvironment may modulate osteoblast activity, proliferation, and differentiation, as well as biosynthetic processes, via the apoptosis pathway. This modulation subsequently impairs osteoblast function and ultimately results in abnormal bone metabolism. These findings provide novel insights into the mechanisms underlying osteoblast apoptosis and osteoporotic diseases, including DOP.

Other bone cells are adversely affected by the diabetic microenvironment. High glucose promoted apoptosis in rat BMSCs through the enhancement of ROS in mitochondria, reduction of mitochondrial membrane potential (MMP), and upregulation of caspase-3/9 and cytochrome C (Cyto C) expression. Maintaining normal MMP is essential for ensuring proper mitochondrial function, including aerobic respiration, energy metabolism, and apoptosis regulation. Elevated levels of ROS within mitochondria can induce the opening of mitochondrial permeability transition pores, resulting in a decrease in MMP and subsequent mitochondrial dysfunction [41]. The investigation demonstrated that high glucose significantly increased the expression of high-mobility group box 1 (HMGB1). Inhibition of HMGB1 was found to enhance the activation of the energy-metabolism-related AMP-activated protein kinase (AMPK) signaling pathway in high-glucose conditions, alleviate mitochondrial dysfunction, and restore the balance of mitochondrial fission and fusion, ultimately leading to a reduction in the apoptosis of BMSCs [42]. Osteocytes are crucial in bone metabolism, as they regulate the functions of osteoblasts and osteoclasts. AGEs can markedly increase the expression of p53 and its downstream target Bax in osteocytes, resulting in heightened activation of caspase-3, thereby promoting apoptosis. Forkhead box O1 (FOXO1), a transcription factor closely associated with apoptosis, has been demonstrated to directly bind to the promoter regions of the apoptosis marker caspase-3 and the osteoblast biosynthesis-related factors sclerostin and RANKL under conditions of elevated AGEs. This binding provides evidence that AGEs induce apoptosis and dysfunction in osteoblasts. The elevated expression of sclerostin and RANKL in osteocytes indicated that AGEs may cause osteocyte dysfunction, leading to reduced osteoblast activity and heightened osteoclast activity, which significantly disrupt bone homeostasis [43]. Thus, the T2DM microenvironment may influence cellular metabolism and biosynthetic processes by modulating the apoptotic process of bone tissue cells, ultimately leading to abnormal bone metabolism and triggering DOP. These studies provide new insights into the increased risk of osteoporosis in diabetes.

## 3. Autophagy

### 3.1. Overview of Autophagy

Cellular autophagy is a dynamic and conserved catabolic process that plays a role in various physiological and pathological processes, including intracellular recycling, nutrient deprivation, and chemoresistance (Table 1) [44,45]. Under physiological conditions, autophagy degrades misfolded proteins and damaged organelles produced by cellular metabolism, facilitating the renewal of specific organelles [46]. Pathological conditions, including nutritional deficiencies, hypoxia, or oxidative stress, lead to an alteration in cellular autophagy to maintain cellular homeostasis [47]. Therefore, increasing evidence suggests that autophagy can also drive cell death in a context-dependent manner (Figure 2) [48].

### 3.2. Autophagy in DOP

Autophagy eliminates damaged organelles and is crucial for sustaining cellular homeostasis [49,50]. In recent years, the role of autophagy in the pathogenesis of DOP has been widely investigated, but the findings remain highly controversial. Jiang et al. reported that prolonged culture of primary mouse osteoblasts in high-glucose conditions led to a significant increase in ROS levels; a substantial decrease in the expression of ALP, Runx2, and OPG; and a marked reduction in calcium nodules. These findings indicate that high glucose severely impairs the osteogenic differentiation and mineralization of osteoblasts. Subsequent investigations revealed that high-glucose-induced dysfunction in osteoblasts is associated with elevated expression of the autophagy-related factor Beclin1. Microtubule-associated protein 1A/1B-light chain 3 (LC3) is one of the autophagy markers. During autophagy, a cytosolic form of LC3 (LC3I) is conjugated to phosphatidylethanolamine to form LC3-phosphatidylethanolamine conjugate (LC3II), which is recruited to autophagosomal membranes. LC3II/LC3I ratio was used to measure the level of autophagy. According to the results, an increased LC3II/LC3I ratio was observed in the high-glucose group. Additionally, there was a significant increase in the number of autolysosomes and autophagosomes, along with a concomitant rise in ROS levels. These results suggest that high glucose may induce osteoblast dysfunction and oxidative stress through the regulation of cellular autophagy [51]. High-glucose-induced autophagy activation was also found in human fetal osteoblasts (hFOB1.19). The expression of autophagy-related proteins, such as LC3-II, PTEN-induced putative kinase 1 (PINK1), and Parkin RBRE3 ubiquitin-protein ligase (Parkin), was increased in hFOB1.19 cells after being cultured in high-glucose medium for several hours. Transmission electron microscopy (TEM) showed that the quantity of mitophagosomes in the high-glucose group was significantly higher than that in the control group. Mitochondrial inhibitors significantly diminished mitochondrial autophagy in the high-glucose group. The expression of osteogenic markers, including OPG, OCN, RUNX2, and Collagen I, was also increased, along with enhanced ALP activity. According to the above results, high glucose may affect the osteogenic capacity of cells by regulating mitochondrial autophagy in osteoblasts, which further leads to the disruption of the bone remodeling balance and causes osteoporosis [52].

Contrary to these results, Wang et al. demonstrated that the expression of the autophagy protein Beclin1 was significantly downregulated in the femurs of GK rats, a well-established T2DM model. Similarly, in vitro studies revealed a significant decrease in Beclin1 levels and the LC3II/LC3I ratio in osteoblasts cultured under high-glucose conditions. These findings suggest that both T2DM and high glucose exposure impair autophagic flux in osteoblasts. Subsequent TEM analyses corroborated these results, showing that high glucose exposure led to a reduction in membranous vacuoles, chromatin condensation in the nucleus, and a decrease in autophagic vacuoles [53]. Mammalian target of rapamycin (mTOR) serves as a central regulator of autophagy [54]. Inhibition of mTOR activates the inhibitor of nuclear factor kappa-B (IκB), which subsequently stimulates the nuclear factor kappa-B (NF-κB) and negatively regulates members of the autophagy regulatory protein family [55,56]. Under high-glucose conditions, mTOR inhibitors were found to counteract the increased expression of LC3, Beclin1, Bax, and Bcl-2 induced by the antioxidant drug timosaponin BII (TBII). These findings suggest that high glucose modulates autophagic activity by upregulating the mTOR/NF-κB pathway, thereby inducing cellular oxidative stress and apoptosis [53]. Another study found that elevated glucose levels increased ROS in MC3T3-E1 cells, reduced the LC3II/LC3I ratio, and suppressed p-ERK expression. These findings suggest that high glucose may alleviate DOP by inhibiting the ROS/ERK-induced autophagy signaling pathway [57]. A clinical study corroborates this finding, showing that BMSCs from T2DM patients demonstrate significantly reduced autophagy levels, an increased senescence phenotype, and impaired osteogenic differentiation capacity. The autophagy of BMSCs can be inhibited by the T2DM microenvironment, and the mechanism may be that insulin promotes premature senescence and inhibits osteogenesis of BMSCs. This may also contribute to bone loss associated with T2DM [58]. These contradictory results suggest that autophagy may have a dual effect on DOP. Mild autophagy may contribute to the preservation of normal cellular status, while a dearth of excessive autophagy may facilitate the development of DOP. The level of autophagy can also be influenced by many factors, such as the type of cell selected. Primary osteoblasts from animals tend to have more complete cell signaling pathways and physiological functions and may be more sensitive to the diabetic microenvironment. In contrast, cell lines such as MC3T3-E1 are not true osteoblasts per se, and certain genes within these cells may have been altered during the process of establishing the cell line. Osteoblastic cell lines are more adaptable to in vitro culture than primary osteoblasts. Furthermore, osteoblasts and BMSCs exhibit significant differences. BMSCs possess a higher differentiation potential and self-renewal capacity compared to osteoblasts, and osteoblasts typically originate from the differentiation of BMSCs [59,60]. Consequently, the regulation of autophagy in BMSCs by the T2DM microenvironment may differ from that in osteoblasts. In addition to cell type, the concentration of glucose in cell models may also influence the extent of cellular autophagy. Recently, it was reported that MC3T3-E1 osteoblasts cultured with fluctuating glucose concentrations produced more ROS than those cultured with constant high glucose, but the monomeric form of JC-1 was slightly decreased, suggesting that mitochondrial damage was not aggravated. Besides, glucose fluctuation significantly increased the protein levels of LC3II and Beclin1 and decreased the expression of sequestosome-1 (SQSTM1, also known as p62). The results of TEM also showed that the number of autophagic vesicles in the glucose fluctuation group was significantly larger than that in the high-glucose group [61]. The aforementioned results suggest that fluctuating glucose concentrations may induce more intense autophagy; however, the underlying mechanism remains to be elucidated. Further experimental studies are necessary to determine whether different glucose concentrations affect the degree of autophagy. In summary, future studies should more carefully distinguish the changes in autophagy and metabolic processes of different kinds of cells in the T2DM microenvironment, as well as the effects of in vitro simulated microenvironments, such as different glucose concentrations, on the degree of cellular autophagy.

The impact of the T2DM microenvironment on various bone cells appears to be inconsistent. Adipose-derived stem cells (ASCs) exhibit significant osteogenic differentiation potential and are frequently utilized in bone tissue engineering [62,63,64]. The expression levels of autophagy-related key factors (Beclin1 and LC3II) and osteogenesis-associated key factors (Runx2 and OPN) were significantly decreased in ASCs from DOP mice cultured in osteogenic induction medium. This finding suggests that T2DM significantly impairs the autophagy level and osteogenic differentiation capacity of ASCs. The autophagic level and osteogenic differentiation ability of DOP ASCs were attenuated upon treatment with autophagy activators. Animal experiments showed that both autophagy and the osteogenic capacity of ASCs were suppressed in DOP mice. Experimental research found that high glucose diminished the osteogenic differentiation potential of ASCs by downregulating autophagy, which subsequently inhibited the Notch signaling pathway [65]. Other research for ASCs revealed that high glucose decreased autophagic flux by blocking autophagosome formation and reducing LC3II expression, which led to a decreased potential for osteogenic differentiation in ASCs. Targeting autophagy enhanced the impaired osteogenic differentiation potential of ASCs in DOP mice [66]. Apart from high glucose, AGEs also reduced the autophagy levels and osteogenic capacity of ASCs. Treatment with the autophagy inducer rapamycin (Rapa) significantly increased the LC3II/LC3I ratio and decreased SQSTM1 expression in ASCs cultured with AGEs, indicating robust activation of autophagy in ASCs. Additionally, Rapa treatment significantly upregulated the expression of Runx2 and OPN, enhanced ALP activity, and increased the number of calcium nodules in ASCs, suggesting restoration of their osteogenic capacity [67]. Collectively, these findings demonstrated that the T2DM microenvironment could modulate the biological processes of ASCs, including proliferation, differentiation, and mineralization, through the regulation of cellular autophagy. Moreover, the T2DM microenvironment influences both osteogenic cell lineages and osteoclasts involved in bone resorption. High glucose was shown to significantly decrease the expression of the osteoclast marker cathepsin K (CTSK) and autophagy-related factors (LC3II and Beclin-1), while simultaneously reducing the number of tartrate-resistant acid phosphatase (TRAP)-positive multinucleated cells. These findings suggest that high glucose downregulates osteoclastogenesis, osteoclast activity, and osteoclast autophagy. Mechanistic investigations revealed that high glucose inhibits the AMPK/mTOR/ULK1 pathway, thereby regulating osteoclast autophagy and further suppressing osteoclastogenesis [68].

## 4. Pyroptosis

### 4.1. Overview of Pyroptosis

The classical pathway of pyroptosis is facilitated by inflammasomes that depend on caspase-1. Inflammasomes are protein complexes composed of germline-encoded pattern recognition receptors (PRRs), apoptosis-associated speck-like protein containing a CARD (ASC), and pro-caspase-1, playing a crucial role in the host defense against various pathogens [12]. The pyroptosis process involves the PPRs, which include NOD-like receptor protein 1(NLRP1), NLRP3, NLR family CARD domain containing 4 (NLRC4), absent in melanoma 2 (AIM2), and Pyrin [69]. PPRs detect pathogen-associated molecular patterns (PAMPs) and damage-associated molecular patterns (DAMPs), leading to their assembly with pro-caspase-1 to form inflammasomes, either in an ASC-dependent or ASC-independent manner, thereby activating caspase-1 [70]. Activated caspase-1 promotes the activation of gasdermin D (GSDMD) and contributes a vital part in the maturation of IL-1β and interleukin-18 (IL-18) precursors (Figure 2) [71]. Upon activation and cleavage, GSDMD can create transmembrane pores in the plasma membrane, which causes the release of excessive inflammatory factors [72]. GSDMD is a crucial molecule that initiates cellular pyrokinesis, significantly contributing to the progression of infectious diseases, tumors, neurodegenerative disorders, and autoimmune diseases (Table 1) [73].

### 4.2. Pyroptosis in DOP

Pyroptosis, or inflammatory death, is facilitated by caspase-1, which activates IL-1β, resulting in its extracellular release in a biologically active form [74]. It has been shown that cellular pyroptosis may be involved in the DOP process by regulating processes such as proliferation and differentiation and the biosynthesis of bone tissue cells. Elevated levels of total ROS, lipid oxidation products, malondialdehyde, and hydrogen peroxide production in BMSCs of T2DM mice have suggested oxidative damage to the cells. Subsequent experiments revealed a notable increase in caspase-1 expression in BMSCs from T2DM mice, alongside elevated serum levels of the inflammatory cytokines IL-1β and IL-18, showing the occurrence of cellular pyroptosis in these BMSCs. Treatment with the ROS scavenger N-Acetyl-L-cysteine (NAC) in both T2DM mice and high-glucose-cultured BMSCs revealed that NAC administration, whether in vivo or in vitro, attenuated the pyroptosis response of BMSCs derived from T2DM mice. Furthermore, NAC enhanced the mineralization of these BMSCs by increasing ALP activity and upregulating the expression of osteogenesis-related markers, including Runx2 and OCN. These effects collectively promoted osteogenic differentiation and accelerated mineralization in BMSCs. The study identified that T2DM-induced pyroptosis elevated IL-1β secretion in BMSCs, subsequently activating osteoclastogenesis and augmenting the resorptive activity of osteoclasts via paracrine-dependent mechanisms. Inhibition of the pyroptosis response in BMSCs significantly decreased the resorptive activity of osteoclasts and downregulated the expression of key osteoclast marker factors, including nuclear factor of activated T cells, cytoplasmic 1 (Nfatc1), CTSK, receptor activator of NF-κB (RANK), and osteoclast stimulatory transmembrane protein (Oc-Stamp). NAC injection in T2DM mice prevented femoral trabecular loss and bone microstructural damage, while enhancing BMD and femoral cortical bone strength. These results revealed that T2DM mice demonstrate a significant ROS-dependent inflammatory response and bone loss. Furthermore, targeted inhibition of the oxidative-damage-dependent pathway could mitigate T2DM-induced skeletal injury and enhance femoral mechanical strength in T2DM mice by promoting the osteogenic differentiation of BMSCs and suppressing osteoclast differentiation and function [75]. Additionally, high glucose can inhibit osteoblast activity and function through the activation of the cellular pyroptosis pathway. The time-dependent decline in MC3T3-E1 cell activity under high-glucose conditions may be attributed to the significant upregulation of the pyroptosis-related proteins caspase-1 and GSDMD, which induce pyroptosis in osteoblasts. Additionally, high glucose inhibits osteoblast differentiation and proliferation via the IL-1β/AKT/β-catenin pathway. The animal experiments showed a significant reduction in the number of osteoblasts in the bone of T2DM mice compared to the normal group, alongside a notable increase in the expression of caspase-1, GSDMD, and IL-1β proteins in the bone. Administration of a pyroptosis inhibitor in T2DM mice resulted in the reversal of the expression of those mentioned pyroptosis-related proteins [76]. In addition to high glucose, AGEs can also cause cellular pyroptosis in bone tissue. Carboxymethyl lysine (CML), the most common type of AGEs, can increase the expression of NLRP3 and cl-caspase-1 through the ORLNC1/miR-200b-3p/Foxo3 signaling pathway, which ultimately leads to apoptosis in mouse BMSCs [77].

In addition to high glucose, T2DM microenvironmental stimuli such as ROS, lipotoxicity, and pro-inflammatory factors also trigger cellular inflammatory responses and even cellular pyroptosis [78]. TNF-α is a significant pro-inflammatory mediator that plays a crucial role in the development of insulin resistance and the pathogenesis of T2DM [79]. TNF-α, primarily synthesized in adipocytes or peripheral tissues, promotes inflammation through the generation of ROS and the activation of transcription factors [80]. The application of TNF-α induced cellular pyroptosis and inhibited the expression of osteoblast marker genes in osteoblasts, as well as ALP activity. Further investigation suggests that TNF-α may inhibit osteoblast differentiation and function in the inflammatory microenvironment via the NLRP3-GSDMD/GSDME pathway [81]. A study for osteoblasts found that TNF-α regulates the expression of hypoxia-inducible factor-1alpha (HIF-1α) and NLRP3 via miR-18a, thus influencing osteoblastic pyroptosis and inhibiting osteogenic differentiation. In vivo studies in T2DM mice indicated that TNF-α suppressed miR-18a expression, thereby activating the HIF-α/NLRP3 axis, which led to osteoclast pyroptosis and hindered osteogenesis [82]. OPG, a member of the tumor necrosis factor receptor superfamily, inhibits osteoclast differentiation and activity by competing with RANKL for binding [83]. OPG promoted the release of IL-1β and IL-18, subsequently inducing osteoclast pyroptosis through the upregulation of NLRP3, ASC, caspase-1, and GSDMD-N [84]. In T2DM condition, oxidative stress results in the accumulation of ROS, which subsequently activates the NLRP3 inflammasome cascade and induces pyroptosis [85]. Induction of oxidative stress via H_2_O_2_ caused a higher level of the inflammation-related NF-κB in osteoblasts. This upregulation of NF-κB triggered the activation of the NLRP3 inflammasome and the production of numerous inflammatory factors, which diminished osteogenic differentiation and mineralization capacity [86].

## 5. Necroptosis

### 5.1. Overview of Necroptosis

Necroptosis is a pro-inflammatory mode of RCD that is governed by receptor-interacting protein kinases (RIPKs) and is independent of caspase family regulation [87]. It is morphologically characterized by fragmented chromatin; swelling of the cytoplasm and organelles, such as lysosomes; and rupture of the cell membrane (Table 1) [88,89]. The necroptosis mechanism primarily involves the cascade activation of receptor-interacting protein kinase 1 (RIPK1), RIPK3, and mixed lineage kinase domain-like protein (MLKL) [90]. Most research has been done on the necrotic pathway that is mediated by TNF-α. Upon TNF-α binding to TNFR, phosphorylated RIPK3 facilitates the recruitment of MLKL, leading to necrosome formation through various biological processes (Figure 2) [91]. Necrosomes ultimately lead to cell membrane rupture and subsequent cell death [92]. Necroptosis has recently been identified as a significant factor in the regulation of various diseases, like tumorigenesis, with necrotic pathways contributing to multiple conditions [93,94]. The following section will discuss the regulation of necroptosis in metabolic diseases related to DOP.

### 5.2. Necroptosis in Osteoporosis and Diabetic Complications

Recent studies indicate a potential association between necroptosis, osteoporosis, and various diabetic complications [95,96]. Cui et al. demonstrated that ovariectomy (OVX) significantly elevated the expression levels of necroptosis markers, including TNF-α, receptor-interacting protein 1 (RIP1), and receptor-interacting protein 3 (RIP3) in rat osteocytes. This indicates that estrogen deficiency resulted in a notable increase in the rates of apoptosis and necroptosis in the osteoblasts of OVX rats [97]. He et al. further investigated the relationship between apoptosis and necroptosis in the osteocytes of OVX rats. The study showed that while both forms of RCD resulted in bone loss, necroptosis exerted a more significant effect on osteoblasts [98]. Furthermore, necroptosis plays a pivotal role in alcohol-induced osteoporosis. Experimental evidence indicates that ethanol consumption promotes necroptosis of osteoblasts within murine femurs, concomitantly impairing osteogenic differentiation and bone formation in vivo. In vitro investigations reveal that ethanol triggers the necroptosis-associated RIPK1/RIPK3/MLKL signaling cascade by augmenting ROS production. This activation subsequently disrupts the osteogenic differentiation and mineralization processes of BMSCs, ultimately leading to apoptosis of BMSCs and contributing to decreased bone mass in mice [99]. Dexamethasone has been shown to induce the activation of RIP1 and its downstream signaling molecules by promoting the production of ROS. Moreover, it inhibits the activity of AMPK, which in turn facilitates the recruitment and activation of RIP3 to RIP1. This cascade of events ultimately leads to necroptosis in osteoblasts [100]. AMPK plays a pivotal role in regulating cellular energy metabolism and has been implicated in various cellular processes, including proliferation, differentiation, and biosynthesis, through the modulation of multiple signaling pathways [101]. The results of this study suggest a close association between the necroptosis of osteoblasts and the regulation of cellular energy metabolism and biosynthesis. Osteoclasts, alongside osteocytes and osteoblasts, play a crucial role in the maintenance of bone homeostasis. The formation of osteoclasts relies on transforming growth-factor-activated kinase 1 (TAK1), with TAK1 deficiency resulting in the spontaneous necrosis of osteoclasts [102]. The treatment of human CD14^+^ monocytes with Smac-mimetics (SM), a compound that inhibits RIPK1 ubiquitination, has been shown to result in a significant reduction in the number of mature osteoclasts, accompanied by a notable decrease in cellular activity and TNF-α-dependent necroptosis. These observations indicate that SM inhibitors may have the potential to selectively decrease the number of osteoclasts and mitigate excessive bone degradation [103]. RIPK3-deficient mice exhibited degradation of trabecular structure and a significant increase in osteoclast numbers, indicating the involvement of RIPK3 in the necroptosis of osteoclasts [104]. Necroptosis of bone cells plays a role in osteoporosis development through the regulation of bone metabolism. The diabetic microenvironment influences the necroptosis process. Necroptosis exerts a considerable effect in diabetic complications, including diabetic nephropathy, diabetic retinopathy, and diabetic cardiomyopathy. Podocytes cultured in high-glucose conditions exhibit elevated levels of RIPK3, pRIPK3, pMLKL, and ROS. Treatment of podocytes with antioxidants and necroptosis inhibitors resulted in decreased expression levels of RIPK3, pRIPK3, and pMLKL, leading to improved podocyte activity [105]. Furthermore, elevated levels of RIPK1, RIPK3, and MLKL were observed in the myocardial tissues of T2DM mice, indicating that high glucose may induce necroptosis in cardiomyocytes [106]. The findings suggest that necroptosis may influence the development of DOP; however, its precise role requires further investigation.

## 6. Ferroptosis

### 6.1. Overview of Ferroptosis

The term “ferroptosis” was coined in 2012 to describe a distinctive form of iron-dependent cell death triggered by the abnormal accumulation of lipid peroxides on cellular membranes [107]. What distinguishes ferroptosis is that it can be blocked by iron chelators or lipophilic antioxidants, not by inhibitors of other common cell death pathways [108]. The main morphological features are mitochondrial atrophy and mitochondrial cristae loss, plasma membrane integrity impairment, organelle swelling, and vesicle formation (Table 1) [107]. Under normal physiological conditions, GPX4 and GSH can reduce the levels of polyunsaturated fatty acids (PUFAs) oxidized by lipoxygenase [109]. When system Xc^−^ is inhibited, decreased GSH synthesis leads to a decrease in the activity of GPX4, which further triggers the accumulation of lipid peroxides, and ultimately leads to cellular ferroptosis [110,111]. The regulation of ferroptosis mainly depends on intracellular iron metabolism and lipid homeostasis [112]. Iron homeostasis is crucial for organismal growth and development, while excess iron induces ferroptosis in cells. Extracellular Fe^3+^, binding with transferrin, can be imported into cytoplasm through transferrin receptor (TFR). Then, intracellular Fe^3+^ is reduced to Fe^2+^ by iron oxide reductase sixtransmembrane epithelial antigen of the prostate 3 (STEAP3) [113]. The reduced form Fe^2+^ is partially transported into a labile iron pool, serving as a source of reactive iron for cells undergoing ferroptosis, while the remainder is stored in ferritin [114]. Ferritin acts as an essential part in ameliorating iron-mediated oxidative damage [115]. Ferritin speeds up ferroptosis through the generation of ROS and ferritinophagy, which is mediated by nuclear receptor coactivator 4 (NCOA4) (Figure 2) [116]. Iron is involved in multiple phases of lipid peroxidation, covering iron-catalyzed lipid oxidation and esterification, the oxidation of PUFAs, and the production of lipid ROS through the Fenton reaction [117].

### 6.2. Ferroptosis in DOP

The progression of DOP is concurrent with abnormalities in glycolipid metabolism; therefore, ferroptosis, which is closely associated with glycolipid metabolism, may be a critical factor in the pathogenesis of DOP. In a T2DM rat model, Lin et al. demonstrated that serum ferritin and TRAP5b (bone resorption marker) levels were significantly elevated, while the expression of ferroptosis markers (SLC7A11 and GPX4) and bone formation markers (ALP and OCN) was significantly decreased. These results indicate that the high-glucose environment of T2DM may induce ferroptosis in osteoblasts, resulting in attenuated osteogenesis and enhanced bone resorption in T2DM rats. After establishing an in vitro model of the T2DM microenvironment, it was determined that high glucose and high fat (HGHF) could regulate the expression of key factors involved in ferroptosis, such as GPX4 and SLC7A11, by activating the METTL3/ASK1-p38 signaling pathway. This activation triggered mitochondrial damage, ultimately leading to the ferroptosis of osteoblasts and impairment of their osteogenic function [118]. The high-glucose microenvironment may directly influence the activity and function of osteoblasts and osteoclasts, resulting in impaired bone remodeling and triggering osteoporosis. Osteoblasts cultured in high-glucose conditions showed increased mitochondrial bilayer density, a decreased number of mitochondrial cristae, accumulation of ROS, and lipid peroxidation. These alterations resulted in oxidative stress and lipid peroxidation in osteoblasts, thereby accelerating cell death [119]. Recent studies have shown that the key pathogenic factor of DOP is the reduced viability of osteocytes. Nonetheless, the mechanisms by which T2DM leads to impaired osteocytic activity and function remain insufficiently explained. Yang et al. demonstrated that the diabetic microenvironment significantly enhanced lipid peroxidation and iron accumulation in osteoblasts, which activated ferroptosis-related pathways, causing ferroptosis and ultimately impaired bone formation [120]. A significant reduction in the expression of GPX4 and SLC7A11, as well as a substantial decrease in the number of osteoblasts in the alveolar bone of mice with diabetic periodontitis, were observed in the research on alveolar bone. In vitro experiments verified that the T2DM microenvironment inhibited the expression of GPX4 and SLC7A11, while elevating inflammatory factor levels in alveolar osteocytes, leading to cellular ferroptosis [26]. A high-glucose culture has been shown to induce ferroptosis in the human osteoblastic cell line hFOB1.19. Du et al. cultured these cells with graded glucose concentrations and demonstrated that high glucose induces ferroptosis in a concentration-dependent manner, thereby attenuating the viability and function of osteoblasts [121]. Similar findings were observed in BMSCs. High-glucose culture significantly elevated ROS and lipid peroxidation levels in mice BMSCs. The antioxidant vitamin K2 restored the osteogenic capacity of BMSCs by modulating the AMPK/SIRT1 signaling pathway, which in turn enhanced the expression of SIRT1, GPX4, and osteogenic markers in the femurs of DOP mice. Additionally, this modulation significantly attenuated ferroptosis in BMSCs and promoted bone formation [122]. The relationship between bone tissue cells and ferroptosis in the diabetic microenvironment requires more studies. A comprehensive investigation of ferroptosis in DOP could reveal potential therapeutic strategies to alleviate oxidative-stress-related cellular damage. Ferroptosis is significantly associated with the pathogenesis of DOP, and its inhibition in bone cells may serve as a potential therapeutic target for DOP treatment.

## 7. Cuproptosis

### 7.1. Overview of Cuproptosis

As a cofactor of various enzymes, copper participates in a variety of physiological and pathological processes such as cellular energy metabolism, mitochondrial respiration, and oxygen transport, and it is one of the indispensable heavy metal elements in the human body [123]. High concentrations of copper perturb mitochondrial homeostasis and induce copper-dependent cell death. Inhibitors of necroptosis, ferroptosis, and oxidative stress are ineffective against this unique cell death, and only copper chelators can inhibit this mode of death [20,124]. The main target of cuproptosis is the TCA cycle, where excess copper bind directly to dihydrolipoyl acetyltransferase (DLAT), contributing to the aberrant oligomerization of acylated proteins, resulting in the loss of the respiratory chain complex Fe-S cluster proteins, which triggers a cellular stress response ultimately leading to cell death (Table 1) [125]. Ferredoxin1 (FDX1), as a key upstream regulator of lipoylated proteins in the TCA cycle, has been found to reduce Cu^2+^ to more toxic Cu^+^ and disrupt the stability of Fe-S cluster proteins [126]. FDX1 is a critical factor in the regulation of cuproptosis. Prolonged exposure to elevated copper levels results in intracellular copper engaging in the Fenton reaction and/or disulfide stress, leading to cell death similar to that observed under anaerobic conditions [127]. The mechanism by which copper carriers induce aggregation and degradation of various mitochondrial proteins remains unclear; however, the main cause of cell death associated with copper carriers is the accumulation of intracellular Cu^+^ (Figure 2) [128]. Similar to other forms of RCD, such as ferroptosis and pyroptosis, cuproptosis has recently been identified as a significant factor influencing the progression of various diseases, including neurodegenerative diseases, cardiovascular diseases, and cancers, all of which are closely associated with copper homeostasis [129]. Both copper deficiency and excessive copper accumulation can negatively affect all tissues. The following section discusses recent findings on cuproptosis within bone metabolism research.

### 7.2. Cuproptosis in Osteoporosis

Cuproptosis is likely to be involved in the dysregulation process of bone metabolism that results in osteoporosis, which is caused by the dysregulation of homeostasis among bone cells [130]. Chen et al. identified 16 common genes by intersecting the differentially expressed genes from bone samples of osteoporosis patients and normal volunteers with the set of cuproptosis-related genes. The WGCNA algorithm was employed to identify genes linked to the cuproptosis phenotype. They subsequently verified the expression of hub genes in the femoral tissues of ovariectomized mice and normal mice through qPCR and immunohistochemistry. The findings showed that these genes exhibited enrichment in pathways related to the inflammatory response, signal transduction, and transcription factor activation, implying that these critical genes in osteoporosis may initiate cuproptosis via the inflammatory response [131]. A separate study utilizing a bioinformatics approach corroborated this conclusion. This study demonstrated differences between osteoporosis and non-osteoporosis samples, confirming that osteoporosis induced alterations in the cuproptosis pathway and elicited an immune response. Further research identified two clusters associated with cuproptosis. Cluster 2 demonstrated increased immunity and notable levels of immune infiltration. This finding indicates that cuproptosis may play a crucial role in the development of osteoporosis and is associated with immune responses [132]. The association between cuproptosis and osteoporosis offers a novel perspective for elucidating the pathogenesis of DOP; however, further studies and experimental data are required to substantiate the role of cuproptosis in DOP.

## 8. Implications of Cell Death Crosstalk

Each RCD exhibits distinct molecular characteristics; however, various forms of RCD share certain common pathways and molecular features, interacting within a complex network of intracellular crosstalk. The interaction between apoptosis and necroptosis mainly involves shared key regulatory molecules, notably tumor necrosis factor receptor 1 (TNFR1) and caspase-8 [133]. A genome-wide association study (GWAS) on the estimation of BMD identified RIPK3 as a potential osteoporosis-related gene [134]. The study further demonstrated that RIPK3 is regulated by TNF-α during necroptosis, a form of programmed necrosis involved in bone resorption. Subsequent investigations revealed that mice deficient in RIPK3 exhibited disorganized bone microarchitecture and an increased number of osteoclasts. These findings suggest that the interplay between osteoclast apoptosis and necroptotic processes may contribute to the development of osteoporosis in mice [104]. AMPK, an energy sensor, plays a crucial role in the regulation of apoptosis and autophagy. The mechanism of multi-drug combinations in tumor treatment involves the induction of mitochondrial dysfunction in tumor cells, which subsequently activates AMPK. The continuous activation of AMPK results in the phosphorylation of Beclin-1, which is then cleaved by caspase-8, ultimately triggering apoptosis in tumor cells. Mutations in Beclin-1 result in the loss of drug-induced phosphorylation of Beclin-1. This abrogation renews autophagic function and suppresses apoptotic processes [135]. Apart from AMPK, ROS also play a pivotal role in modulating both apoptosis and autophagy [136,137]. A high-glucose environment can enhance ROS production, exacerbate oxidative stress, and impair mitochondrial function, ultimately contributing to various complications associated with T2DM [138,139,140]. Meanwhile, ROS production can induce apoptosis in various cell types, including pancreatic β-cells, endothelial cells, and osteoblasts [141,142,143]. Zhang et al. further investigated the effects of hyperglycemia on apoptosis and autophagy in MC3T3-E1 osteoblasts. Their results demonstrated that hyperglycemia significantly reduced osteoblast proliferation and activated autophagy. The underlying mechanism may involve hyperglycemia-induced ROS production, declined mitochondrial membrane potential, and subsequent upregulation of autophagy markers such as LC3II and apoptosis-associated factors such as Bax and caspase-3, ultimately leading to the induction of apoptosis and autophagy in osteoblasts. When the antioxidant NAC was applied, ROS levels in osteoblasts were significantly reduced, autophagy-related protein expression was inhibited, and osteoblast apoptosis was mitigated [61]. These findings suggest that autophagy and apoptosis may interact via ROS formation in the T2DM microenvironment.

Moreover, RIPK1, a key factor in necroptosis, is involved in the regulation of autophagy [144]. Impaired autophagic flux promotes the activation of RIPK1 and MLKL, which in turn affects the process of necroptosis [145]. In an in vitro model of diabetic cardiomyopathy, HGHF culture conditions activate the necroptosis-associated RIPK1/RIPK3 pathway and upregulate the expression of autophagy-related proteins LC3-II and p62, thereby inducing myocardial fibrosis and impairing both diastolic and systolic cardiac functions. Inhibition of RIPK1 or RIPK3 expression restores impaired autophagic flux and mitigates HGHF-induced cardiac fibroblast death and fibrosis [146]. These findings indicate that HGHF conditions contribute to cardiomyocyte dysfunction through the activation of necroptosis and autophagy signaling pathways. The interaction between autophagy and ferroptosis is mainly through ferritinophagy, and the specific mechanism is that NCOA4-mediated ferritinophagy leads to excessive release of iron, which ultimately induces the Fenton reaction and accelerates the ferroptosis process [147,148]. Ferroptosis and ferritinophagy have been reported in both the brains of diabetic mice and in neuronal cells cultured under high-glucose treatment, as evidenced by decreased the expression of GPX4, SLC7A11, and ferritin, and increased levels of NCOA4. Further mechanistic studies revealed that neuronal function was improved following the inhibition of ferroptosis and ferritinophagy using melatonin [149]. In addition to NCOA4, oxidative stress markers such as ROS can also modulate the interplay between ferroptosis and autophagy. High-glucose culture significantly elevated levels of lipid peroxides, ROS, and GPX4 in hFOB1.19 cells, while increasing the number of autophagosomes and autolysosomes, and upregulating the expression of autophagy-related proteins. After the treatment of oxidative stress inhibitor NAC, intracellular ROS levels and autophagy activity were significantly reduced [121].

There may also be an interaction between pyroptosis and apoptosis. A study on anti-inflammatory and antioxidant drugs demonstrated that both pancreatic and renal tissues were damaged in a T2DM rat model induced by fructose combined with nicotinamide and STZ. In the kidneys, the expression of the oxidative stress marker 4-hydroxynonenal (4-HNE), the pyroptosis marker IL-1β, the autophagy marker LC3II, and the apoptosis marker caspase-3 were significantly elevated. Similarly, caspase-3 and LC3II levels were significantly elevated in the pancreas of T2DM rats, with caspase-3 expression being markedly higher than that of LC3II. This suggests that apoptosis may be more pronounced than autophagy in pancreatic β-cells of T2DM rats. When antioxidant and anti-inflammatory drugs were administered, all of the above indicators were significantly alleviated [150]. These findings indicate that the T2DM microenvironment may induce apoptosis, pyroptosis, and autophagy in different tissues, with these RCD processes collectively contributing to tissue dysfunction and disease progression in T2DM. However, the mechanisms underlying RCD interactions require further investigation, and the specific mechanisms of this crosstalk and interactions in bone tissue cells during DOP development need to be supported by additional research evidence.

PANoptosis is a unique lytic and inflammatory cell death pathway regulated by the PANoptosome complexes, which have the characteristics of apoptosis, pyroptosis, and necroptosis at the same time but cannot be solely accounted for by any of the three RCD pathways [151]. Previous studies have shown that the activation of pyroptosis, apoptosis, and necroptosis in the process of PANoptosis is regulated by a common cell-death-inducing complex [152]. Malireddi et al. found that when TAK1 was absent, apoptosis, pyroptosis, and necroptosis were activated in a RIPK1-independent manner [153]. The above finding suggests that these complexes (PANoptosomes) are flexible skeletons in which core components of different cell death pathways will be recruited to perform RCD. PANoptosis has been shown to be intricately linked to the inflammatory processes and immune responses in bone tissue cells and immune cells, and it plays a role in the pathogenesis of osteoporosis [154]. However, the precise “sensors” that initiate PANoptosis in DOP remain to be fully characterized, and further investigation is required to elucidate the specific roles of these key factors and the signaling networks implicated in the progression of DOP.

While interactions among various RCD pathways have been documented, there is a conspicuous lack of investigation on such synergistic effects within bone tissue. Given the complexity of the pathogenesis of DOP and the precision of regulation among bone cells, it is extremely challenging to determine the occurrence mechanism of each RCD and the impact of the interactions among different RCD on the development of DOP. This is a significant issue that requires attention in future research.

## 9. Therapeutic Implication of Cell Death in DOP

### 9.1. Clinical Drugs

Clinical medications used to treat T2DM also have effects on bone tissue. Metformin, sulfonylureas, glucagon-like peptide-1 receptor activator, and dipeptidyl peptidase-4 inhibitors have moderate or mild protective effects on bone tissue [155,156]. In contrast, thiazolidinediones and sodium-glucose cotransporter protein 2 inhibitors increase fracture risk [157,158] Studies have reported that glibenclamide (Gli) can rescue osteoblast apoptosis induced by high glucose, restore cell proliferation rate, and improve the mineralization of osteoblasts [159]. It is hypothesized that sulfonylureas may contribute to the delay of bone loss through the inhibition of apoptosis in bone cells. Empagliflozin is a selective sodium-glucose cotransporter protein-2 (SGLT-2) inhibitor that mitigates diabetic myocardial microvascular injury and acute kidney injury [160,161]. ASCs from DOP mice were reported to have attenuated osteogenic differentiation potential and autophagic activity, while the use of empagliflozin could enhance autophagic flux by promoting autophagosome formation and autolysosome acidification, resulting in increased LC3II expression and decreased SQSTM1 level, ultimately improving ASCs dysfunction caused by high glucose. Furthermore, empagliflozin contributed to the reversal of osteogenic inhibition induced by the diabetic microenvironment in ASCs. The protective effect of empagliflozin on ASCs in DOP mice was eliminated when autophagy activity was inhibited by 3-methyladenine (3-MA). The findings indicate that the enhanced autophagic activity triggered by empagliflozin may significantly promote the osteogenic differentiation of ASCs derived from DOP mice [66]. The above results indicate that drugs used for T2DM are likely to play a protective effect on bone tissue by modulating the RCD pathway.

Irbesartan, utilized clinically for hypertension management, has shown a protective effect on bone health in recent studies. Irbesartan markedly reduced oxidative stress and RAGE expression induced by AGEs in osteoblasts, subsequently decreasing osteoblastic apoptosis. Irbesartan treatment in db/db mice significantly decreased ROS level and apoptosis in bone tissues, resulting in improved trabecular microarchitecture and enhanced biomechanical properties of the bone. This indicates that irbesartan may have a protective function against diabetes-related bone damage by inhibiting the oxidative stress and apoptotic effects mediated by AGEs/RAGE pathway [162].

### 9.2. Natural Products

Besides the clinical drugs, some natural products also affect the RCD process in bone tissue (Table 2). Puerarin has received widespread attention for its superior bioactivity and antioxidant properties [163]. Treatment with puerarin of osteoblasts cultured with high glucose inhibited the expression of caspase-3/8/9 and pro-apoptotic factors, while increasing the expression of bone formation markers such as ALP and Runx2 and decreasing the levels of bone resorption markers. Puerarin significantly reduced inflammation and apoptosis in osteoblasts. Similarly, puerarin treatment significantly inhibited T2DM-induced cellular inflammation and apoptosis in bone tissues, reduced bone loss, and attenuated microstructural damage of bone tissue, as well as lowered serum glucose levels and alleviated insulin resistance in T2DM rats. The application of puerarin to inhibit apoptosis and mitigate cellular dysfunction in bone tissue presents a potential strategy for the prevention and treatment of DOP [36]. Silibinin is a natural compound characterized by its antioxidant properties. Silibinin was found to significantly reduce RAGE expression and further regulate AGEs-induced osteoblast apoptosis by modulating RAGE-mediated mitochondrial pathways [164]. Icariin has the capacity to mitigate the decline in mitochondrial membrane potential and dysfunction caused by iron overload. It reduces intracellular ROS levels, which enhances osteogenesis and inhibits osteoclasts [165]. Astragalus polysaccharide reduced ROS accumulation in BMSCs and inhibits apoptosis [166]. Quercetin might significantly inhibit erastin-induced ferroptosis by reducing lipid peroxides and total ROS in BMSCs [167]. TBII, the major steroidal saponin constituent from the rhizome of Anemarrhena asphodeloides Bunge, could upregulate Beclin1 expression, while attenuating autophagic fluxes and delaying osteoblast injury by high glucose. Oral administration of TBII resulted in improved tibial microstructure, decreased phosphorylation levels of mTOR and NF-κB, and increased expression levels of Beclin1 in the tibia of DOP rats. This finding implies that TBII attenuates high-glucose-induced oxidative stress and apoptosis by inhibiting the osteoblast mTOR/NF-κB signaling pathway to activate autophagy [53]. Natural products derived from various dietary ingredients and medicinal plants can modulate RCD through diverse mechanisms, demonstrating significant potential for the prevention and treatment of DOP. This finding will hopefully help in the development of complementary and alternative medicines characterized by low cost, minimal side effects, and suitability for long-term use.

### 9.3. Cell Death Inhibitors

Numerous cell death signaling pathways have been progressively identified through the comprehensive examination of the RCD process. The significant role of RCD in the pathogenic process of DOP suggests that synthetic or natural products influencing key regulators in cell-death-related pathways could serve as effective treatments for DOP. Preclinical studies indicate the efficacy of certain cell death inhibitors in addressing orthopedic diseases.

Necroptosis is a caspase-family-independent regulation of RCD that is associated with the activation of RIP1/3. Necrostatin-1 (Nec-1, an inhibitor of RIP1 activity) has been demonstrated to be a specific inhibitor of necroptosis and to play an important role in diseases such as chronic kidney disease, ischemic brain injury, and myocardial infarction [168,169,170]. Recent studies indicate that Nec-1 contributes to glucocorticoid-induced osteoporosis by promoting bone cell activity and enhancing bone formation in rats [171]. Comparable effects have been found in OVX rats. Nec-1 inhibited necroptosis in osteoblasts resulting from estrogen deficiency by reducing the expression of TNF-α, RIP1, and RIP3, which led to decreased bone turnover and enhanced bone microarchitecture [97]. Additionally, Nec-1 can inhibit alcohol-induced bone loss by reducing RIPK1 kinase activity, which subsequently inhibits the RIPK1/RIPK3/MLKL axis, leading to a decrease in osteoblast necroptosis [99].

Hydroxychloroquine (HCQ) is a widely utilized clinical immunomodulator that significantly influences various diseases through the modulation of autophagy-related pathways [172]. Recent studies indicate that HCQ significantly inhibits the accumulation of lipid peroxides and ROS in osteoblasts induced by high glucose, while also preventing the degradation of GPX4. The findings suggest that HCQ may enhance osteoblast viability and function through the modulation of cellular redox homeostasis and the reduction of cellular autophagy [121]. NAC can inhibit oxidative stress and mitochondrial damage induced by high glucose in osteoblasts, and modulate autophagic activity and ferroptosis, thereby providing a protective effect on osteoblasts [173]. A widely recognized autophagy inhibitor, 3-MA, diminishes autophagy activity and ferroptosis by targeting the AMPK/mTOR/ULK1 pathway, thereby mitigating high-glucose-induced autophagy in osteoclasts, which subsequently influences osteoclast formation and function [68]. Additionally, vitamin D has been shown to exert a regulatory effect on autophagy. The high-glucose culture resulted in lower osteoblastic activity and a notable increase in ROS level in mice, simultaneously promoting heightened autophagic activity in osteoblasts. Vitamin D reduced the accumulation of ROS induced by high glucose and activated the PI3K/Akt signaling pathway. The activation led to the inhibition of FOXO1 expression, and then decreasing of the excessive autophagy induced by prolonged high-glucose culture and increasing of the expression of osteogenic markers. The above results suggest that vitamin D can reduce high-glucose-induced autophagy, alleviate osteoblast dysfunction and intracellular oxidative stress by regulating the PI3K/Akt/FOXO1 pathway, and thus attenuate the damage of bone tissue in mice by T2DM [51]. Torin1 functions as an autophagy activator by targeting mTOR [174]. The autophagy level and osteogenic differentiation capacity of ASCs in DOP mice were inhibited. Following the administration of the autophagy activator Torin1, which targets mTOR, the viability of ASCs in DOP mice improved, accompanied by an increase in the protein expression levels of Beclin1 and LC3II. In parallel, the expressions of Runx2 and OPN was found to be upregulated in a concentration-dependent manner with respect to Torin1 [175]. The results reveal that Torin1 may enhance the osteogenic differentiation capacity of ASCs derived from DOP mice by elevating their autophagy levels.

Iron chelators exhibit beneficial therapeutic effects in orthopedic diseases due to iron overload. The mechanism by which deferoxamine (DFO) treats osteoporosis may involve the reduction of HIF expression in bone tissue and the activation of the downstream Wnt/β-catenin signaling pathway, thereby accelerating bone formation [176]. The activated Wnt/β-catenin signaling pathway concurrently promotes the differentiation of BMSCs into osteoblasts [177]. Radiation induced a decrease in hepcidin activity and an increase in iron levels in mice. The injection of DFO eliminated the iron-caused deterioration of bone trabecular microstructure in irradiated mice. Meanwhile, DFO was found to inhibit osteoclast differentiation in in vitro experiments, suggesting that lowering the iron level may be one of the targets for the effective treatment of osteoporosis [177].

Necrosulfonamide (NSA), a pyroptosis inhibitor, has been shown in studies to reverse the cell activity decline and cellular pyroptosis induced by adenosine triphosphate (ATP) and lipopolysaccharide (LPS). It also enhanced the expression levels of pyroptosis-related factors. Inhibiting the secretion of inflammatory factors interleukin 6 (IL-6), TNF-α, and IL-1β in osteoblasts reversed the effects of ATP/LPS on ALP activity and expression of differentiation-related genes in osteoblasts. The overexpression of caspase-1, GSDMD, and NLRP3 abolished the effects of NSA on the viability and pyroptosis of osteoblasts, and decreased the expression of osteoblast-differentiation-related genes and ALP activity, indicating that NSA promoted the proliferation and differentiation of osteoblasts by inhibiting NLRP3/caspase-1/GSDMD pathway [178]. The above findings provide evidence for the potential application of NSA in improving the fracture repair function of osteoblasts and suggest the value of the NLRP3/caspase-1/GSDMD focal death pathway as a drug target.

## 10. Future Perspectives and Conclusions

**Figure 3:**
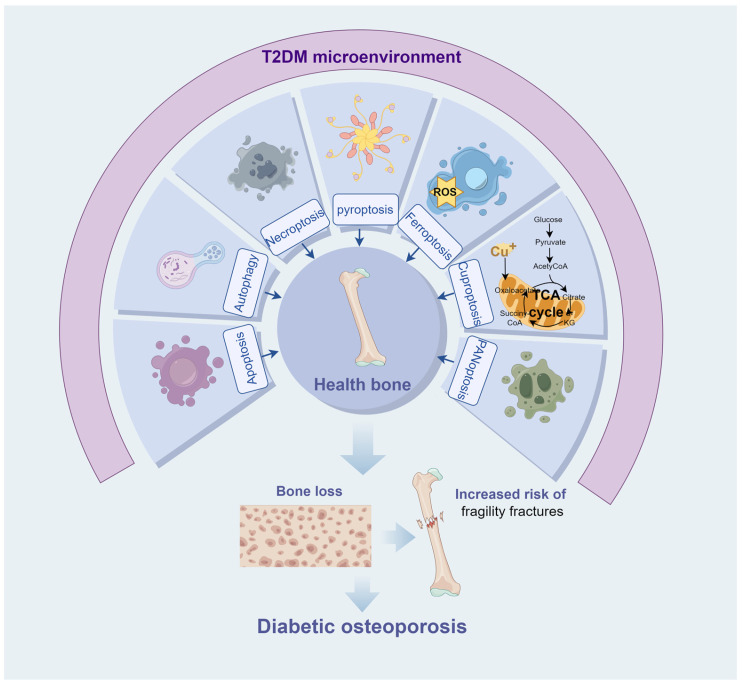
Forms of RCD in diabetic osteoporosis.

DM has caused significant distress and complications for both patients and clinicians. T2DM is susceptible to numerous consequences, with DOP being one of the prevalent ones. DOP is latent and can be life-threatening in severe instances due to complications in fracture healing following DOP formation. Consequently, it is necessary to investigate the molecular mechanism of DOP. This review summarizes recent advancements regarding the RCD mechanism to enhance DOP (Figure 3). There are still certain perspectives in this field that warrant further examination. First, various types of RCD contribute to the pathogenesis of DOP. Current studies have primarily focused on the roles of apoptosis and autophagy in DOP, while other types of RCD have received less attention. This may be attributed to the earlier discovery and more intensive study of apoptosis and autophagy compared to others. Apoptosis and autophagy likely play a more significant role in T2DM-induced bone loss compared to other forms of RCD. Consequently, additional research is required to clarify the role of various RCD types in DOP. Second, the form of a dominant RCD and the interactions among various forms of RCD in DOP are still unclear. Different forms of RCD are interdependent and may affect the fate of bone cells through cooperation. Future investigations must elucidate the interrelationships and dominance of various RCD in DOP to uncover the multifactorial regulatory mechanisms governing death forms during DOP development.

Recent studies have focused on method selection for targeting RCD in the treatment of DOP, highlighting the necessity of identifying specific mechanisms to elucidate the relationship between RCD and DOP. Simultaneously, identifying RCD-related markers at various stages and in different cells of DOP represents a viable preventive strategy. Bone, as a specialized connective tissue, exhibits different anatomical forms, including long, short, flat, and irregular bones, which serve essential functions in the support and protection of the human body. Furthermore, bone exhibits specific hematopoietic, endocrine, and immune functions. The forms and roles of RCD in different regions of bone tissue during DOP and how they vary during DOP development are not well studied. Further clarification of the relationship between intercellular communication and RCD will enhance the understanding of the RCD mechanism in DOP. Current studies on targeting drugs for RCD have primarily concentrated on a single type of death. Therefore, exploring drugs that can simultaneously target and regulate multiple RCD types, or the combination of drugs affecting different RCD types with potential synergistic effects, may be a new direction in the search for improved therapeutic approaches for DOP.

This review summarizes the roles of various forms of RCD in DOP, aiming to elucidate the molecular mechanisms underlying T2DM-induced bone loss. In diabetic bone tissues, RCD such as apoptosis, autophagy, pyroptosis, and ferroptosis can modify the activity and function of bone cells. Consequently, bone remodeling and metabolic processes are impaired in diabetic pathological conditions, resulting in bone loss and heightened risk of fragility fracture. Despite the discovery of a multitude of natural compounds and pharmaceutical agents that possess the ability to induce or regulate RCD and exhibit remarkable therapeutic potential, the existing experimental evidence available to corroborate the efficacy of these substances remains scarce and inconsistent. Further research into the relationship between RCD and DOP will facilitate the identification of more effective therapies in the near future.

## Figures and Tables

**Table 1:** Characteristics of the major types of regulated cell death.

Type	Morphological Features	Biochemical Features	Key Regulator Molecules	Pores	Major Inhibitors (Target)	Key Pathways
Apoptosis	Shrinkage, plasma membrane blebbing, nucleus condensation and rupture, chromatin condensation	Formation of apoptotic bodies, caspase activation, cleavage of caspase substrates, Δψm dissipation, Fas-FasL pathway, TNF-α/TNFR1 pathway	Death receptors and their ligands, Bax, Bak, Bcl-2, AIF, caspase-3, caspase-8, caspase-9, p53	Extrinsic (No) Intrinsic (Mito outer)	Z-VAD-FMK, Z-VAD(OH)-FMK (pancaspase), Z-DEVD-FMK (caspase-3, -6, -7, and -10), Z-VDVAD-FMK (caspase-2), ivachtin (caspase-3), Ac-DEVD-CHO (caspase-3 and-7), Z-IETD-FMK (caspase-8), Q-LEHD-OPh (caspase-9)	TNFR P53/Bcl-2 NF-κB Apaf-1/Cyto C
Autophagy	Autophagic vacuolization	Caspase-independent, LC3 lipidation, formation of autophagosome, elevated autophagic flux, and lysosomal activity	AMPK, mTOR, ULK1, PI3KIII, BECN1, LC3	No	Chloroquine (lysosome), bafilomycin A1, concanamycin A (H^+^-ATPase), 3-methyladenine, wortmannin (PI3K), spautin 1(USP10 and USP13)	ULK complex PI3K/Akt/mTOR Ras/Raf/MAPKs Beclin-1 p62
Pyroptosis	Cells gradually flattening, pore formation on cells’ membranes, rupture and bubbling of plasma membranes, moderate chromatin condensation	Caspase-dependent, gasdermin cleavage, formation of inflammasome, IL-18 and IL-1β release	NLRs, ALRs, caspase-1, caspase-11, GSDMD	Plasma membrane	Disulfiram, LDC7559, Ac-FLTD-CMK, Polyphyllin VI (GSDMD), morroniside (MMP2/9)	Caspase-1/4/5/11-GSDMD Caspase-3/GSDME
Necroptosis	Cells swelling, swelling of cytoplasmic organelles, nuclear condensation (pyknosis), plasma membranes rupture, and chromatin fragmented	Caspase-independent, RIPK1/RIPK3-mediated phosphorylation of MLKL	Death receptors, TLRs, TCR, RIPKs, MLKL	Plasma membrane	Tetrahydroisoquinolines, lactoferrin, DNase (NETs), cl-amidine (PADI4)	RIPK1/RIPK3/MLKL TLR4/TICAM1 ZBP1
Ferroptosis	Cells smaller and rounder, normal nuclear size, pore formation on cells membranes, smaller mitochondria, decreased mitochondria crista, and elevated mitochondrial membrane densities	Caspase-independent, iron accumulation, lipid peroxidation, system Xc^−^/GSH/GPX4 pathway inhibition	System Xc^−^, GPX4, lipid ROS	No	Deferoxamine, ciclopirox, deferiprone (Fe), ferrostatin-1, liproxstatin-1, β-mercaptoethanol, vitamin E, β-carotene, NAC, CoQ10, baicalein (ROS), vildagliptin, alogliptin, linagliptin (DPP4), thiazolidinedione, rosiglitazone (ACSL4), selenium (GPX4)	ACSL4/LPCAT3/ALOX15 SLC7A11/GPX4/NFE2L2
Cuproptosis	Mitochondrial dysfunction	Elesclomol-induced, Cu-dependent, Cu(I) binds to lipoylated components of TCA cycle, destabilization of Fe–S cluster proteins	SLC31A1, ATP7B, FDX1	No	GSH (chelate Cu), UK5099 (MPC), rotenone, antimycin A (ETC)	SLC31A1/ATOX1/ATP7A SLC31A1/COX17/COX11 CCS/SOD1

Abbreviations: ACSL4 (acyl-CoA synthetase long chain family member 4); AIF (apoptosis-inducing factor); Akt (protein kinase B); ALOX15 (lipoxygenase-15); ALRs (melanoma-2-like receptors); AMPK (AMP-activated protein kinase); Apaf1 (apoptotic protease-activating factor-1); ATOX1 (antioxidant 1); ATP7B (ATPase copper-transporting beta); Bcl-2 (B-cell lymphoma 2); BECN1 (beclin 1); Bak (Bcl-2 homologous antagonist/killer); Bax (Bcl-2-associated X); CCS (copper chaperone for superoxide dismutase); COX11 (cytochrome c oxidase copper chaperone COX11); COX17 (cytochrome c oxidase copper chaperone COX17); CoQ10 (coenzyme Q10); Cyto C (cytochrome C); DPP4 (dipeptidyl peptidase-4); ETC (electron transport chain); Fas (tumor necrosis factor receptor superfamily, member 6); FasL (Fas Ligand); FDX1 (Ferredoxin1); GSH (glutathione); GPX4 (glutathione peroxidase 4); GSDMD (gasdermin D); IL-1β (interleukin-1beta); IL-18 (interleukin-18); LC3 (microtubule-associated protein 1A/1B-light chain 3); LPCAT3 (Lysophosphatidylcholine acyltransferase 3); MAPK (mitogen-activated protein kinase); MLKL (mixed lineage kinase domain-like protein); MMP (matrix metalloproteinase); MPC (mitochondrial pyruvate carrier); mTOR (mammalian target of rapamycin); NETs (neutrophil extracellular traps); NFE2L2 (nuclear factor, erythroid 2 like 2); NLRs (nucleotide-binding and oligomerization domain-like receptors); NF-κB (nuclear factor kappa B); PADI4 (protein-arginine deiminase type-4); PI3K (phosphatidylinositol 3-kinase); p62 (sequestosome 1, SQSTM1/p62); Ras (rat sarcoma); Raf (rapidly accelerated fibrosarcoma); RIPK1 (receptor-interacting protein kinase 1); RIPK3 (receptor-interacting protein kinase 3); ROS (reactive oxygen species); SLC31A1 (solute carrier family 31 member 1); SLC7A11 (solute carrier family 7 member 11); SOD1 (superoxide dismutase); TCA (tricarboxylic acid); TCR (T cell receptor); TICAM1 (toll-like receptor adaptor molecule 1); TLRs (toll-like receptors); TNF-α (tumor necrosis factor-alpha); TNFR1 (TNF receptor superfamily member 1A); TP53/p53 (tumor protein p53); ULK1 (unc-51 like autophagy-activating kinase 1); USP (ubiquitin-specific peptidase); ZBP1 (Z-DNA-binding protein 1).

**Table 2:** Natural products for clinical use by targeting RCD.

Natural Products	Sources	Structure	Clinical Adverse Drug Reactions/Events	Pharmacological Properties
Astragalus polysaccharide	Dry root of Astragalus membranaceus	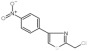	Widely used in endocrine system diseases	Immunomodulation, anti-tumor, and antioxidant
Icariin	Most copious constituent in Herba Epimedii	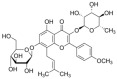	Widely used clinically, beneficial in osteoporosis, chronic nephritis, asthma, hepatitis, and cardiovascular problems	Reduction of inflammation, antioxidant effect, anticancer, and anti-aging activities
Puerarin	Roots of Pueraria lobata (Willd.) Ohwi.	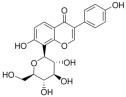	Widely used clinically, mainly seen in immune system and hematologic adverse reactions	Antioxidant, anticancer, anti-inflammation, promoting bone formation, attenuating insulin resistance
Quercetin	Various foods such as apples, berries, red onions, and tea (Camellia sinensis)	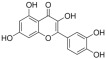	Widely used in the treatment of cancer, allergic reactions, inflammation, arthritis, and cardiovascular disorders	Possesses antioxidant properties and protects against oxidative stress
Silibinin	Foremost active compound extracted from Silybum marianum	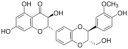	Widely utilized as a therapeutic agent for liver disease	Anti-oxidant, anti-apoptosis, anti-inflammation, anti-cancer, and many more
Timosaponin BII (TBII)	Anemarrhena asphodeloides Bunge	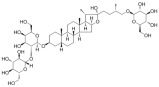	Developed as a pharmaceutical for prevention or treatment of dementia	Neuroprotection, enhancement of learning and memory, vascular protection and inhibition of platelet aggregation

## Data Availability

Not applicable.
